# Reporter cell assay for human CD33 validated by specific antibodies and human iPSC-derived microglia

**DOI:** 10.1038/s41598-021-92434-2

**Published:** 2021-06-29

**Authors:** Jannis Wißfeld, Mona Mathews, Omar Mossad, Paola Picardi, Alessandro Cinti, Loredana Redaelli, Laurent Pradier, Oliver Brüstle, Harald Neumann

**Affiliations:** 1 Institute of Reconstructive Neurobiology, Medical Faculty, University of Bonn and University Hospital of Bonn, Venusberg-Campus 1, 53127 Bonn, Germany; 2 Axxam SpA, Via Meucci 3, 20091 Bresso, Italy; 3 Sanofi, 1 Av P. Brossolette, 91380 Chilly-Mazarin, France; 4 Cellomics Unit, LIFE & BRAIN GmbH, Venusberg-Campus 1, 53127 Bonn, Germany

**Keywords:** Alzheimer's disease, Drug screening, Microglial cells, Neuroimmunology, Innate immunity

## Abstract

CD33/Sialic acid-binding Ig-like lectin 3 (SIGLEC3) is an innate immune receptor expressed on myeloid cells and mediates inhibitory signaling via tyrosine phosphatases. Variants of CD33 are associated with Alzheimer’s disease (AD) suggesting that modulation of CD33 signaling might be beneficial in AD. Hence, there is an urgent need for reliable cellular CD33 reporter systems. Therefore, we generated a CD33 reporter cell line expressing a fusion protein consisting of the extracellular domain of either human full-length CD33 (CD33M) or the AD-protective variant CD33^ΔE2^ (D2-CD33/CD33m) linked to TYRO protein tyrosine kinase binding protein (TYROBP/DAP12) to investigate possible ligands and antibodies for modulation of CD33 signaling. Application of the CD33-specific antibodies P67.6 and 1c7/1 to the CD33M-DAP12 reporter cells resulted in increased phosphorylation of the kinase SYK, which is downstream of DAP12. CD33M-DAP12 but not CD33^ΔE2^-DAP12 expressing reporter cells showed increased intracellular calcium levels upon treatment with CD33 antibody P67.6 and partially for 1c7/1. Furthermore, stimulation of human induced pluripotent stem cell-derived microglia with the CD33 antibodies P67.6 or 1c7/1 directly counteracted the triggering receptor expressed on myeloid cells 2 (TREM2)-induced phosphorylation of SYK and decreased the phagocytic uptake of bacterial particles. Thus, the developed reporter system confirmed CD33 pathway activation by CD33 antibody clones P67.6 and 1c7/1. In addition, data showed that phosphorylation of SYK by TREM2 activation and phagocytosis of bacterial particles can be directly antagonized by CD33 signaling.

## Introduction

CD33/Sialic acid-binding Ig-like lectin-3 (SIGLEC3) is an innate immune receptor expressed on the cell surface of myeloid cells and is composed of an IgV domain, a C2 domain and a single-pass transmembrane domain followed by an immunoreceptor tyrosine-based inhibitory motif (ITIM) and an ITIM-like domain^1,2^. Recently, a polymorphic allele of CD33 (variant rs3865444(A)) was found to be negatively correlated with the risk to develop Alzheimer’s disease (AD), thus, being AD-protective for the carrier^3,4^. This CD33 variant is co-inherited with the CD33 variant rs12459419(T), which modulates the splicing efficiency of exon 2 in CD33^4^. Exon 2 partially encodes for the IgV domain of CD33, which mediates sialic acid binding. Therefore, CD33 lacking exon 2 (D2-CD33/CD33^ΔE2^) is missing the functional sialic acid binding domain. Additionally, this CD33^ΔE2^ variant was found to show a reduction of CD33 surface expression on microglia^3,5^. In line with these observations, functional analyses showed that CD33 expression levels positively correlate with the amount of Aβ and Aβ plaque load in the brains of AD patients, while patients expressing the CD33^ΔE2^ variant exhibit decreased amyloid-β deposition in the brain^3^.

CD33 mediates inhibitory signaling via the ITIM domain and thus, inhibits cellular activation and proliferation, including cytokine production and phagocytosis^6–8^. Phosphorylation of the ITIM and ITIM-like domains of CD33 by Src family tyrosine kinases lead to recruitment and activation of phosphatases such as Src homology region 2 (SH2) domain-containing protein tyrosine phosphatase (SHP) 1 and 2 or SH2-containig inositol phosphatase 1 (SHIP1)^6,9^. These phosphatases are capable to counteract proinflammatory signaling originating from immunoreceptor tyrosine-based activating motifs (ITAMs), such as the triggering receptor expressed on myeloid cells 2 (TREM2)-associated TYROBP/DAP12. Both, TREM2 and TYROBP were also linked to AD^10,11^. Consequently, there appears to be a direct crosstalk between CD33 and TREM2 in AD^12^, but evidence for direct signaling interference between TREM2 and CD33 in a human cellular system is still inconclusive*.* Studying CD33 signaling is hampered by the fact that it has very short and transient kinetics. After interaction of the receptor with an appropriate ligand, the intracellular ITIM domain is first phosphorylated by membrane-associated Src kinases and then recruits phosphatases (SHP1, SHP2 or SHIP1) that lead to dephosphorylation of the ITIM domain itself and theoretically of microcluster-associated ITAM-domains of TYROBP/DAP12, too^7,13,14^. Accordingly, cellular systems to study signaling of the human cell surface receptor CD33 that are also suitable for high-throughput drug screening are rare or unavailable.

Here, we established a human cell-based reporter system for CD33 by fusing the extracellular domain of full-length CD33 (CD33M) or CD33^ΔE2^ to TYROBP/DAP12. Using phosphorylation of SYK (pSYK) and calcium imaging as readouts we confirmed that the two putative agonistic CD33 antibodies, clone P67.6 and clone 1c7/1, were able to activate CD33 signaling. Furthermore, CD33 antibody clones P67.6 and 1c7/1 were able to antagonize the TREM2-triggered increase in pSYK and decreased the phagocytic uptake of bacterial particles in human induced pluripotent stem cell-derived microglia.

## Results

### Generation of human CD33 full-length (CD33M) and variant CD33^∆E2^ reporter cell lines

**Figure 1:**
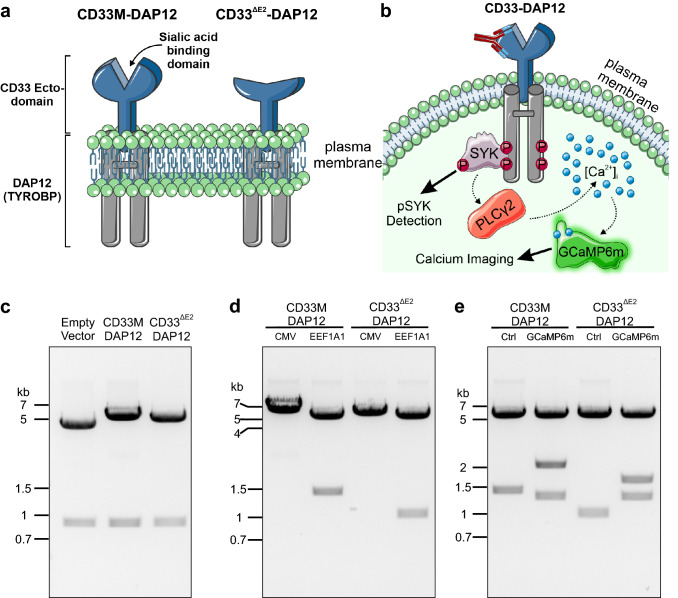
CD33 reporter cell line constructs. (**a**) Schematic drawing of the two CD33-DAP12 constructs. Both, the full CD33 ecto-domain (CD33M) and the ecto-domain lacking the sialic acid binding domain (CD33^ΔE2^) were fused to TYROBP/DAP12. (**b**) Schematic drawing of the readouts for the CD33 reporter cell line. CD33 can be activated by ligands or antibodies, which results in increased SYK phosphorylation and consequently increased intracellular calcium levels. (**c**) Gel electrophoresis image of CD33-DAP12 constructs cloned into pcDNA5/FRT after digestion by EcoRI. (**d**) Successful exchange of the viral CMV promoter with the human *EEF1A1* promoter was indicated by a second band after digestion with XhoI at 1,339 bp (CD33M) or 958 bp (CD33^ΔE2^). (**e**) Gel electrophoresis image of pcDNA5/FRT-CD33-DAP12-GCaMP6m plasmids after digestion with XhoI. GCaMP6m positive clones exhibited three bands compared to the control (Ctrl) with only two bands. Gel images were cropped for better visualization. Supplementary Fig. 1 shows the uncropped full-length gel.

A chimeric CD33-DAP12 strategy was applied to shift the transient CD33 signaling from inhibitory to activatory (Fig. 1a). The transmembrane and intracellular parts of CD33 were replaced by the human TYROBP/DAP12 containing a point mutation (p.D50A) to eliminate possible interactions with activatory receptors such as TREM2. Reporter cell lines for both, the full-length CD33M and the variant CD33^∆E2^ lacking the sialic acid binding side were generated (Fig. 1a). Signaling of CD33 was detected as phosphorylation of SYK by the AlphaLISA system and imaging of calcium fluxes by a calcium-sensitive green fluorescent protein (GFP) variant, GCaMP6m (Fig. 1b).

Successful cloning of the two CD33 variants, CD33M and CD33^∆E2^, fused to DAP12 into the pcDNA5/FRT vector was demonstrated by restriction digestion with EcoRI (Fig. 1c). As predicted, CD33M-DAP12 positive clones showed two bands at 5,227 and 883 bp, which was clearly distinguishable from CD33^∆E2^-DAP12 positive clones (4,846 and 883 bp) and the empty vector as control (4,195 and 883 bp). To prevent loss of expression of the protein of interest over time, particularly by methylation of the viral CMV promoter^15,16^, the promoter of the pcDNA5/FRT plasmid was successfully exchanged by the human *EEF1A1* promoter, in both CD33-DAP12 constructs and the empty vector. Thereby, successful introduction of *hEEF1A1* was indicated by an additional band at 1339 bp (CD33M) or 958 bp (CD33^ΔE2^) after restriction digestion with XhoI (Fig. 1d).

In addition, two more constructs were cloned, in which a calcium-sensitive green fluorescent protein (GFP) variant, GCaMP6m, was introduced into the plasmid for improved live cell calcium imaging (Fig. 1e). The GCaMP6m gene was separated from the CD33-DAP12 construct via an internal ribosomal entry site (IRES), which enables independent translation of the two proteins. Subsequently, all plasmids were validated via Sanger sequencing.

Thus, four stable reporter cell lines, two for analysis of SYK phosphorylation (CD33M-DAP12, CD33^∆E2^-DAP12) and additional two for analysis of intracellular calcium fluxes (CD33M-DAP12-GCaMP6m and CD33^∆E2^-DAP12-GCaMP6m) were generated.

### Expression of CD33 on the cell surface of the reporter cell lines

**Figure 2:**
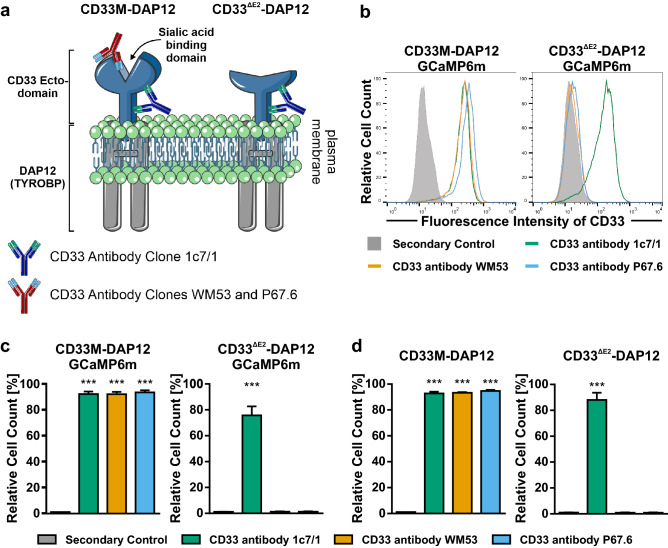
Flow cytometric analysis of CD33 surface expression. (**a**) Schematic drawing of the CD33-DAP12 constructs. Both, the full CD33 ecto-domain (CD33M) and the ecto-domain lacking the sialic acid binding domain (CD33^∆E2^) were fused to TYROBP/DAP12. CD33^∆E2^ can be identified by binding of the antibody clone 1c7/1 (blue) but not WM53 or P67.6 (red), whereas all three antibody clones can bind CD33M. (**b**) The CD33-DAP12 and CD33-DAP12-GCaMP6m cells were stained for CD33 surface expression with the antibody clones 1c7/1, WM53 and P67.6. A representative flow cytometry histogram plot for the CD33M-DAP12-GCaMP6m cells is shown (left side). All three tested antibodies were able to stain full-length CD33 on the cell surface. Expression of variant 2 CD33 from CD33^∆E2^-DAP12 and CD33^∆E2^-DAP12-GCaMP6m cells was only detected by antibody clone 1c7/1. A representative flow cytometry histogram plot for the CD33^∆E2^-DAP12-GCaMP6m cells is shown (right side). (**c**) Quantification of CD33 staining showed a high percentage of CD33 expressing cells in the CD33M-DAP12-GCaMP6m line for all three tested antibody clones but only the CD33 antibody clone 1c7/1 was able to detect CD33 in CD33^∆E2^-DAP12-GCaMP6m expressing cells. The antibody clones WM53 and P67.6 did not show any staining of CD33^∆E2^-DAP12 expressing cells. (**d**) Quantification of CD33 staining revealed a high percentage of cells in the CD33M-DAP12 line expressed CD33, and was detected by all three antibody clones. CD33 in CD33^ΔE2^-DAP12 expressing cells was only detected by antibody clone 1c7/1. Data are shown as mean + SEM of three to five independent experiments; *** *p* ≤ 0.001 compared to Secondary Control determined by Welch ANOVA followed by Games-Howell post hoc test.

Validated CD33 plasmids were stably transfected into Flp-In-293 cells using the Flp-In/FRT system. To distinguish between the full-length CD33M-DAP12 and the CD33^∆E2^-DAP12 variant, which lacks the sialic acid binding V-set domain^4,17^, the CD33 antibody clones 1c7/1, WM53 and P67.6 were used (Table 1). Antibody 1c7/1 is able to identify both constructs as it binds an epitope in the constant C2-set Ig-like domain, whereas WM53 and P67.6 can only stain the full-length CD33M-DAP12 expressing cells because they bind an epitope proximal of the sialic acid binding domain in the V-set Ig-like domain (Fig. 2a). Cell surface expression of full-length CD33M and variant CD33^∆E2^ was measured by flow cytometry. CD33M-DAP12-GCaMP6m expressing lines showed high extracellular CD33 staining. All three tested antibody clones (WM53, P67.6 and 1c7/1) detected the full-length CD33 on the cell surface (Fig. 2b + c). In detail, the CD33 antibody clones 1c7/1, WM53 and P67.6 exhibited 91.53% ± 1.64%, 93.72% ± 1.55% and 92.42% ± 1.89% positive cells, respectively (*p* < 0.001 for each antibody compared to control). Detection of variant 2 CD33 on the cell surface of the CD33^∆E2^-DAP12-GCaMP6m lines was dependent on the applied antibody clone. The two antibody clones WM53 and P67.6, which bind the variable V-set domain that is partially missing in the CD33^∆E2^ gene did not show any staining (1.29% ± 0.23% and 1.32% ± 0.28%, respectively; Fig. 2b + c). However, variant 2 CD33 expression (from the CD33^∆E2^ gene) was detected by 1c7/1 with 75.72% ± 6.93% (*p* < 0.001 compared to antibody control). Likewise, CD33M-DAP12 reporter cell showed high CD33 surface expression detected by 1c7/1 (92.80% ± 1.30%, *p* < 0.001), WM53 (93.27% ± 0.50%, *p* < 0.001) or P67.6 (94.63% ± 0.82%, *p* < 0.001) and CD33^∆E2^ was only recognized by 1c7/1 (88.03% ± 5.51%, *p* < 0.001) on the cell surface of CD33^∆E2^-DAP12 expressing cells (Fig. 2d).

**Table 1:** CD33-specific and control antibodies.

Antibody/target	Clone	Reactivity	Host	Manufacturer	Catalogue #/details	Used in which assay?
CD33	P67.6	Human	Mouse	Santa-Cruz Biotechnology	sc-19660	Calcium imaging
	P67.6	Human	Mouse	BioLegend	366602	Phagocytosis and pSYK
	P67.6 F(ab)	Human	Mouse	Sanofi	F(ab) fragment of P67.6	All
	1C7/1	Human	Mouse	Cedarlane	7CL7627AP	All
	WM53	Human	Mouse	Southern-Biotech	9590–01	All
Isotype	IgG1	–	Mouse	Abcam	18437	All
Isotype	IgG F(ab’)2	–	Mouse	Thermo Fisher Scientific	31203	All

Thus, the CD33M-DAP12 and CD33M-DAP12-GCaMP6m as well as the CD33^∆E2^-DAP12 and CD33^∆E2^-DAP12-GCaMP6m reporter cell lines expressed CD33 or CD33^∆E2^, respectively, on the cell surface.

### Putative agonistic CD33-specific antibodies increased phosphorylation of SYK in the full-length CD33 expressing reporter cell line

**Figure 3:**
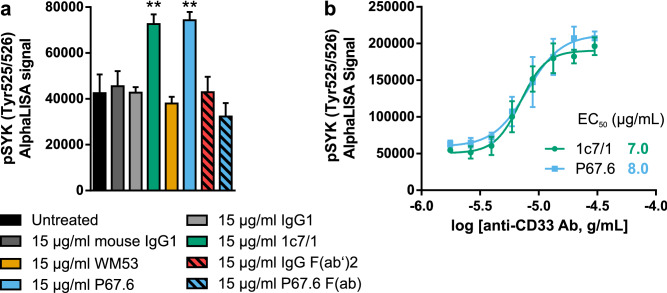
pSYK detection in CD33M-DAP12 cell lines. (**a**) pSYK detection in CD33M-DAP12 reporter cells treated with CD33-specific antibodies. Addition of CD33 antibodies P67.6 and 1c7/1 resulted in increased pSYK levels, whilst P67.6 F(ab), WM53 as well as the different isotype IgG1 control antibodies did not show any change in endogenous pSYK levels. (**b**) 1c7/1 and P67.6 dose–response curve in CD33M-DAP12 reporter cells. Addition of CD33 antibody clones 1c7/1 and P67.6 resulted in an increase in endogenous pSYK levels measured 30 min after the treatment. Data are presented as mean ± or + SD; ** *p* ≤ 0.01 compared to untreated determined by one-way ANOVA analysis followed by Dunnett’s post hoc test.

After confirmation of CD33 cell surface expression, we evaluated the endogenous phosphorylation levels of SYK (pSYK, Tyr525/526) through AlphaLISA technology upon CD33 stimulation, using the CD33M-DAP12 cell line. In particular, we tested four CD33-specific antibodies (clones 1c7/1, WM53, P67.6 and P67.6 F(ab)) at 15 µg/ml (Fig. 3a). The P67.6 and 1c7/1 antibody clones were able to functionally activate the chimeric CD33M-DAP12 receptor with the subsequent downstream activation of SYK in its activation loop, at the Tyr525/526 phosphorylation site^18^. Interestingly, the monovalent F(ab) fragment of P67.6 and CD33 antibody clone WM53 were inactive. Further, we determined the EC_50_ of the two CD33 activating antibodies 1c7/1 and P67.6. Analysis of the dose–response relationship resulted in an EC_50_ of 7–8 µg/ml for both antibody clones (Fig. 3b).

In summary, measurement of endogenous pSYK levels suggested the activation of the full-length CD33M-DAP12 signaling pathway by the CD33 antibody clones P67.6 and 1c7/1, supporting an agonistic mode of action.

### CD33-specific antibodies induced signaling and led to increased intracellular calcium levels in the full-length CD33 expressing reporter cell line

**Figure 4:**
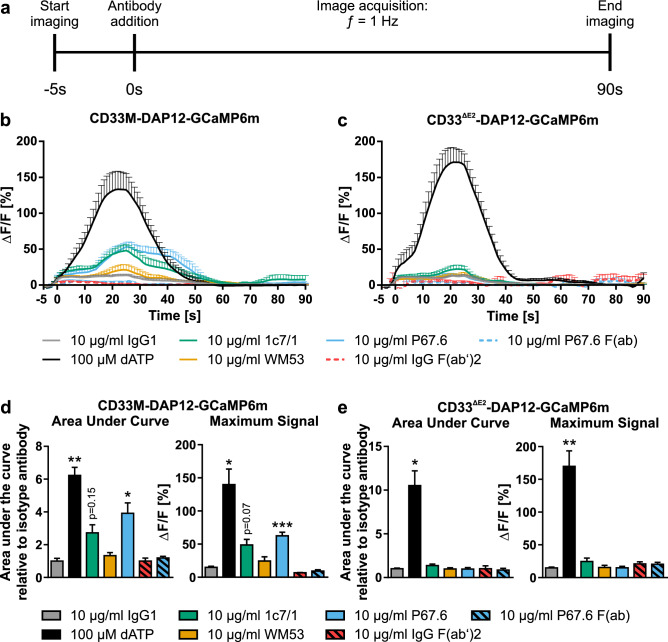
Calcium imaging in CD33-DAP12-GCaMP6m reporter cell lines. (**a**) Schematic time line of image acquisition and compound handling. (**b**, **c**) Calcium imaging analyzed as *ΔF/F(t)* in CD33-DAP12-GCaMP6m lines. Addition 100 µM dATP led to a strong increase in intracellular calcium levels in both cell lines, CD33M- and CD33^∆E2^-DAP12-GCaMP6m, with a peak at around 20–25 s. The CD33 antibody clones 1c7/1 and P67.6 evoked a selective intracellular calcium response only in CD33M-DAP12-GCaMP6m cells. The CD33 antibody clones WM53 and P67.6 F(ab) as well as the isotype IgG1/F(ab’)2 antibodies did not show a change in intracellular calcium levels. (**d**, **e**) The area under the curve as well as the maximum *ΔF/F(t)* signal calculated from independent experiments showed a significant increase in dATP treated samples in both CD33-DAP12-GCaMP6m lines and a selective increase in CD33M-DAP12-GCaMP6m expressing cells if treated with the CD33 antibody clone P67.6 or 1c7/1. Data are presented mean + SEM; n = 3–6; *** *p* ≤ 0.001, ** *p* ≤ 0.01, * *p* ≤ 0.05 compared to 10 µg/ml IgG1 determined by Welch ANOVA followed by Games-Howell post hoc test.

To further validate the putative CD33 agonistic antibodies, the responses of the CD33M-DAP12-GCaMP6m and CD33^∆E2^-DAP12-GCaMP6m cell lines to our panel of CD33-specific antibodies were analyzed by imaging of the intracellular calcium flux (Fig. 4). The activation of the CD33 receptor should result in activation of SYK and PI3K/PLCγ2 via its artificially linked TYROBP/DAP12^19–22^. Subsequently, PI3K/PLCγ2 should lead to IP_3_ generation and thus increase the intracellular calcium from the endoplasmic reticulum (ER) and other organelles (Fig. 1b). Further, dATP was used as a positive control. Extracellular dATP leads to an increase in intracellular calcium levels via the purinergic P2 receptor family and PI3K/PLCγ2^23,24^.

Image acquisition was performed with ƒ = 1 Hz and for 95 s with 5 s of baseline recording (Fig. 4a). For this assay, we adjusted the treatment concentration to 10 µg/ml, which is close to the EC_50_ previously determined. The isotype antibody IgG1 did not show a remarkable response in both cell lines. However, addition of 100 µM dATP led to an increase in the relative fluorescence intensity in both cell lines as demonstrated by changes in the area under curve (AUC) and maximum *ΔF/F(t)* signal. In detail, the maximum *ΔF/F(t)* signal in CD33M-DAP12-GCaMP6m expressing cells was 139.76% ± 22.04% (*p* = 0.011) and in CD33^∆E2^-DAP12-GCaMP6m expressing cells 169.67% ± 23.78% (*p* = 0.006; Fig. 4b–e). Similarly, the AUC calculated for the treatment with dATP showed 6.22 ± 0.49 (CD33M-DAP12-GCaMP6m, *p* = 0.002) versus 10.51 ± 1.69-fold change (CD33^∆E2^-DAP12-GCaMP6m, *p* = 0.03) compared to the control (Fig. 4d,e). Further, an increase in intracellular calcium transients for the CD33M-DAP12-GCaMP6m expressing cells was observed after addition of the CD33-specific antibody P67.6, whereas the antibodies WM53 and P67.6 F(ab) did not lead to a notable response. In detail, the antibody P67.6 evoked an increase in relative fluorescence intensity with a 3.92 ± 0.64-fold change in AUC (*p* = 0.04) compared to the control IgG1 and a maximum *ΔF/F(t)* signal of 62.57% ± 4.91% (*p* < 0.001). Addition of 10 µg/ml 1c7/1 led to a similar but not significant increase in intracellular calcium levels with an area under the curve of 2.72 ± 0.49-fold change (*p* = 0.15) compared to IgG1 and a maximum *ΔF/F(t)* signal of 48.70% ± 7.66% (*p* = 0.07; Fig. 4b–e). The CD33^∆E2^-DAP12-GCaMP6m expressing cell line did not show an increase in intracellular calcium levels for any of the tested antibodies (Fig. 4c–e).

Thus, the CD33-DAP12 cell lines provide a reliable tool for testing CD33-specific antibodies. Further, the CD33-specific antibody clone P67.6 was confirmed to act agonistic on full-length CD33 fused to DAP12. The CD33-specific antibody clone 1c7/1 tended to have a similar agonistic effect on full-length CD33 fused to DAP12, but without statistical significance.

### CD33-specific antibody clones P67.6 and 1c7/1 also acted agonistic in human iPSC-derived microglia

**Figure 5:**
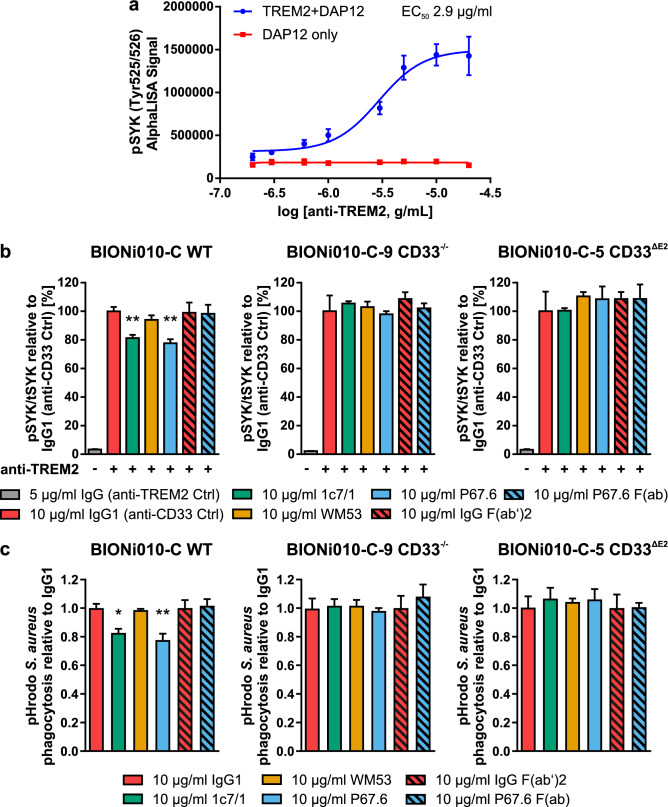
Activation of endogenous CD33 in iPSdMiG by CD33 agonistic antibodies. (**a**) pSYK analysis in TREM2 + DAP12 reporter cells. Addition of anti-TREM2 antibody AF1828 resulted in an increase in endogenous pSYK levels measured 30 min after the treatment only in TREM2 + DAP12 but not DAP12 expressing control reporter cells. Data are presented mean ± SD. (**b**) CD33 antibodies P67.6 and 1c7/1 were able to decrease the increased pSYK/tSYK levels triggered by TREM2 activation in WT iPSdMiG (left). In CD33^−/−^ (middle) and CD33^ΔE2^ (right) iPSdMiG none of the tested antibodies was able to modulate pSYK/tSYK levels after TREM2 activation. Data are presented mean + SEM; n = 3–6; ** *p* ≤ 0.01 compared to IgG1 (anti-CD33 Ctrl) determined by Welch ANOVA followed by Games-Howell post hoc test. (**c**) CD33 antibodies P67.6 and 1c7/1 decreased the phagocytic uptake of pHrodo *S. aureus* BioParticles in WT iPSdMiG (left). In CD33^−/−^ (middle) and CD33^ΔE2^ (right) iPSdMiG none of the tested antibodies was able to modulate pHrodo *S. aureus* BioParticle phagocytosis. Data are presented mean + SEM; n = 3; ** *p* ≤ 0.01 and * *p* ≤ 0.05 compared to IgG1 determined by ANOVA followed by Dunnett’s post hoc test.

After confirmation of CD33 activation by the antibody clones P67.6 and 1c7/1 in two different assay systems by using the CD33-DAP12 and CD33-DAP12-GCaMP6m reporter cell lines, we evaluated the activity of these antibodies in human induced pluripotent stem cell-derived microglia (iPSdMiG). To that end we obtained human iPSdMiG from wild type (WT/CD33M), CD33 knockout (CD33^−/−^) and CD33^∆E2^ (D2-CD33/CD33m) isogenic induced pluripotent stem cells. IPSdMiG expressed typical lineage-specific markers including CD11b, CD45, CD64, CD68, CX3CR1, IBA1, P2RY12, PU.1 and TMEM119 identified by immunocytochemical staining (Fig. S2a). Further, CD33M gene transcription and CD33 surface expression were sharply decreased or even completely absent in CD33^−/−^ and CD33^∆E2^ iPSdMiG compared to WT CD33M expressing iPSdMiG. However, CD33^∆E2^ iPSdMiG showed increased gene transcript levels of the CD33^∆E2^ isoform compared to WT and CD33^−/−^ iPSdMiG using variant-specific primers spanning exon-exon junction 1/3 (Fig. S2b–d). In iPSdMiG endogenous ITIM signaling originating from e.g. CD33 might counterbalance activatory ITAM signaling. Therefore, we co-stimulated iPSdMiG with a TREM2 activating antibody (AF1828; Table 2), which was first validated in TREM2 + DAP12 reporter cells with an EC_50_ of 2.9 µg/ml (Fig. 5a). Treatment of iPSdMiG for 5 min with the stimulatory TREM2 antibody AF1828 increased phosphorylation of SYK, as determined by the AlphaLISA assay. Co-stimulation with 10 µg/ml of the CD33 antibody clones P67.6 or 1c7/1 decreased pSYK levels in wild type CD33M expressing iPSdMiG from 100% ± 3.06% to 76.79% ± 2.75% (*p* = 0.01) or 81.24% ± 2.35% (*p* = 0.004), respectively (Fig. 5b left). Interestingly, this effect was not observed for CD33 antibody clones WM53 or P67.6 F(ab) and completely absent in CD33^−/−^ and CD33^∆E2^ iPSdMiG (Fig. 5b middle and right). Similarly, stimulation of WT iPSdMiG with 10 µg/ml of the CD33-specific antibodies P67.6 and 1c7/1 but not WM53 or P67.6 F(ab) decreased the phagocytic uptake of pHrodo-labeled *S. aureus* compared to the isotype control. In detail, anti-CD33 clone P67.6 dampened pHrodo-labeled *S. aureus* uptake from 1.00 ± 0.03 to 0.78 ± 0.04 (*p* = 0.007) and clone 1c7/1 to 0.83 ± 0.03 (*p* = 0.03) in WT CD33M expressing iPSdMiG (Fig. 5c left). Again, this effect was not visible for CD33 antibody clones WM53 or P67.6 F(ab) and none of the tested antibody clones exhibited an effect in CD33^−/−^ and CD33^∆E2^ iPSdMiG (Fig. 5c middle and right).

**Table 2:** TREM2-specific and control antibodies.

Antibody/target	Clone	Reactivity	Host	Manufacturer	Catalogue #/details	Used in which assay?
TREM2	Polyclonal	Human	Goat	R&D Systems	AF1828	pSYK
Isotype	Polyclonal	–	Goat	Abcam	ab224187	pSYK

Thus, the CD33 antibody clones P67.6 and 1c7/1 are able to activate CD33 as well as modulate TREM2 signaling at the level of SYK phosphorylation and decrease the phagocytic uptake of *S. aureus* BioParticles.

## Discussion

Recently, several genome-wide association studies (GWAS) linked polymorphisms in the CD33 gene to Alzheimer’s disease (AD)^25,26^. The CD33 SNP rs3865444(C) has been associated with increased risk to develop AD^27^, while the less common allele for the CD33 SNP rs3865444(A) was found to decrease the risk to develop AD^25,26^. Thus, therapeutic targeting of CD33 by antibodies might be beneficial in AD. However, development of CD33-interfering drugs was hampered by the lack of appropriate models for studying human CD33 in cellular systems.

In the present study, we developed a human cell-based CD33 reporter system, which was used to confirm two putative agonistic CD33-specific antibodies (clones 1c7/1 and P67.6). Furthermore, the effect of these antibodies on CD33 signaling was validated in an orthogonal assay using human induced pluripotent stem cell-derived microglia. Here, we showed that the agonistic CD33 antibodies directly counteracted the TREM2-triggered phosphorylation of SYK and decreased the phagocytic uptake of pHrodo-labeled *S. aureus* BioParticles. Interestingly, the AD-protective CD33 variant with the SNP rs3865444(A) was found to be co-inherited with rs12459419(T), which mediates exon 2 splicing^4^. The exon 2 of CD33 encodes partially for the IgV sialic acid-binding domain. We therefore used CD33 lacking exon 2 (variant CD33^∆E2^/D2-CD33) as control to evaluate our cellular reporter system.

The SIGLEC receptor CD33 shows strong species differences between mouse and human, since SIGLECs evolved very quickly in humans generating several orthologs without direct corresponding homologs in the mouse. In humans, CD33 predominantly signals via intracellular ITIM but not via the ITIM-like domain^6,7,28^, whereas murine CD33 only bears an intracellular ITIM-like, but no ITIM domain. Further, murine CD33 has a positive charged residue in its transmembrane domain, which is known to be critical for interaction with ITAM-containing proteins such as TYROBP/DAP12^2,22^. A previous study also highlighted the functional differences between human and murine CD33^29^. Deletion of human CD33, but not murine CD33, led to decreased phagocytosis in macrophages and microglia. Further, cell surface expression of murine CD33 was entirely dependent on murine DAP12 expression, which was not described for human CD33^29^. Thus, murine CD33 does not reflect the human situation and requires the development of human-based systems to study CD33 signaling. Of note, human CD33 signaling shows bidirectional kinetics with fast and transient tyrosine phosphorylation of the ITIM by Src family kinases, followed by Src homology-2-containing tyrosine phosphatase 1 (SHP1) and SHP2, which bind to phosphorylated CD33 and dephosphorylate the ITIMs in an autoregulatory manner as well as ITAM-associated signaling molecules^30^. Furthermore, recruitment of SHP1 to the intracellular CD33 domain triggers endocytosis of the CD33 receptor^30^. Interestingly, inhibitory NK cell receptor signaling could be redirected to activatory signaling by replacing the ITIM domain with ITAM-containing signaling chains^31^. This approach was also used in genetic engraftment of a tumor-specific chimeric antigen receptor (CAR) in NK cells and has been tested in vitro to compare the ITAM-containing CD3ζ signaling chain with the ITAM-containing DAP12 chain that even showed increased efficiency^31^.

In the present study, we used a similar approach to overcome the limitations of human CD33 signaling and created a chimeric human CD33-DAP12 reporter system for both CD33M and CD33^∆E2^. Therefore, we replaced the transmembrane and the intracellular inhibitory domains of human CD33M and CD33^∆E2^ with the corresponding activatory TYROBP/DAP12 domain, in which we mutated the charged residue in the transmembrane domain to avoid association with ITAM-signaling receptors. These constructs were stable transfected into Flp-In-293 cells, an engineered human embryonic kidney cell line. Using this concept, we circumvented measurement of the highly variable and transient ITIM phosphorylation or SHP1/2 recruitment. However, it needs to be considered that this cellular model system is only able to identify ligands or allosteric activators of CD33 binding to the extracellular domain of CD33. Modulators of the intracellular ITIM domain, which also might have the potential to antagonize ITAM signaling cannot be detected. Therefore, this model system is specific for studying extracellular CD33 modulators.

Remarkably, both constructs CD33M-DAP12 and CD33^∆E2^-DAP12 were detected on the plasma membrane using flow cytometry with the CD33 antibody clone 1c7/1, which binds an epitope in the C2 domain of CD33. Thus, CD33^∆E2^ was not translocated into peroxisomes in our CD33^∆E2^-DAP12 cell lines, as previously described for blood neutrophils and monocytes^5^. Surface expression of CD33^∆E2^-DAP12 in the reporter cell lines might be attributed to the direct interaction with DAP12, which is already known to stabilize murine CD33 surface expression^29^. Flow cytometric analysis using the CD33 antibody clones WM53 and P67.6 showed CD33 surface expression only in CD33M-DAP12 expressing cells. This is in line with the literature since both antibody clones bind an epitope within the IgV sialic acid-binding domain, which is missing in CD33^∆E2^ due to splicing of exon 2^4^.

On the molecular level, activation of CD33 in the CD33-DAP12 reporter cell lines results in an ITAM-mediated cellular response. Phosphorylation of DAP12 by Src kinases leads to recruitment and phosphorylation of SYK, which further results in an increase in intracellular calcium levels in a PI3K-PLCγ2-dependent manner^19–21^. Here, we used both measurements, namely intracellular calcium fluxes as well as SYK phosphorylation, as readouts to identify agonistic CD33-specific antibodies. The transient increase in intracellular calcium levels was observed only in CD33M-DAP12, but not in CD33^∆E2^-DAP12 expressing cells upon stimulation with CD33 antibodies 1c7/1 and P67.6. Interestingly, the F(ab) version of P67.6 was not able to stimulate the CD33M-DAP12 reporter cell line suggesting a need for crosslinking of the receptor to enable downstream signaling. The agonistic effect of the two CD33 antibodies 1c7/1 and P67.6 was also demonstrated by their ability to increase the phosphorylation of SYK in CD33M-DAP12 expressing cells. In contrast, WM53 had no agonistic effect underlining key functional differences between these CD33-specific antibodies.

However, the approach of a chimeric CD33-DAP12 receptor introduced into a non-immune cell has the limitation that it lacks the recruitment of phosphatases during CD33 signaling. To test whether the two identified agonistic CD33 antibody clones (1c7/1 and P67.6) were capable to activate endogenously expressed native CD33, we used human induced pluripotent stem cell-derived microglia as a model system. IPSdMiG expressed typical lineage-specific markers CD11b, CD45, CD64, CD68, CX3CR1, IBA1, P2RY12, PU.1 and TMEM119. Moreover, CD33 surface expression was only detected in CD33M expressing WT but not in CD33^−/−^ or CD33^∆E2^ iPSdMiG in line with recent findings^5^. In human macrophages and microglia, CD33 theoretically counteracts an immune response originating from ITAM signaling. Phosphorylation of SYK was increased in this microglia model after activation of the ITAM-associated receptor TREM2 by an agonistic antibody. Co-treatment with the agonistic CD33 antibodies 1c7/1 or P67.6 dampened the phosphorylation of SYK. This modulation of SYK phosphorylation was not observed in CD33^−/−^ and CD33^∆E2^ microglia. Likewise, CD33 antibody clones 1c7/1 and P67.6 were able to decrease the phagocytic uptake of *S. aureus* bacterial particles in WT but not CD33^−/−^ and CD33^∆E2^ microglia. Interestingly, antibody concentrations of approximately the IC_50_ value determined in the CD33 reporter cell line were only able to attenuate the TREM2-triggered increase of SYK phosphorylation by 20–30%. Thus, it might be possible that the capacity of CD33 to modulate TREM2 signaling is limited due to its autoregulatory nature or that TREM2 is more abundant on the cell surface than CD33, so that higher antibody concentrations would be needed to show a similar activation as in the reporter cell line. In addition, the exact epitope of these antibodies is not known to date. Thus, a small molecule directly targeting the ligand binding domain of CD33 principally might be more effective in attenuating TREM2 signaling than these antibodies.

Taken together, our data show that the novel chimeric CD33-DAP12 reporter cell line we developed can be used to study CD33 activation. We showed that two of the tested CD33-specific antibodies (1c7/1 and P67.6) were capable to activate CD33 in the reporter cell lines as well as in human iPSC-derived microglia. Furthermore, we showed a direct modulation of TREM2 signaling by agonistic activation of CD33 in human iPSC-derived microglia. Thus, the system can be used to identify further agonistic CD33-specific antibodies as well as small molecule modulators binding to CD33.

## Methods

### Culture of Flp-In-293 cells

Flp-In-293 cells (R75007, Thermo Fisher Scientific) and its derivatives were cultured according to an adapted version of the manufacturer’s instructions. Briefly, frozen cells were thawed in pre-warmed 293 medium (DMEM + L-Glutamine and 4.5 g/l D-glucose, 10% fetal bovine serum (FBS), 2 mM L-glutamine, 1 mM sodium pyruvate, 0.1 mM NEAA (Gibco)). Consequently, Flp-In-293 cells were cultivated in 293 medium containing 100 µg/ml Zeocin. Constitutive expression cell lines were cultured in 293 medium + 150 µg/ml Hygromycin B. The cells were passaged when reaching 80–90% confluency by detaching chemically using 0.25% Trypsin/EDTA.

### Generation of CD33 reporter cell lines

The full mRNA sequence of human CD33/SIGLEC3 and human TYROBP/DAP12 were obtained from NCBI (Gene IDs 945 and 7305, respectively). The CD33-DAP12 fusion protein lacks the intracellular ITIM and the transmembrane domains, which were exchanged by the TYROBP/DAP12 sequence representing an ITAM domain. Further, a point mutation was introduced into the DAP12 gene (p.D50A), which eliminates possible interactions with TREM2, by using site-directed mutagenesis (#A13282, Thermo Fisher Scientific) following the manufacturer’s instructions. Two different CD33-DAP12 constructs were generated: CD33M-DAP12 and CD33^∆E2^-DAP12, which lacks the sialic binding domain (D2-CD33/CD33m; Fig. 1a). Moreover, the genetically encoded calcium indicator (GECI) GCaMP6m^32^ was introduced into CD33M- and CD33^∆E2^-DAP12 plasmids separated via an internal ribosomal entry site (IRES) motif. GCaMP6m was purchased as pGP-CMV-GCaMP6m from Addgene (Plasmid #40754). In addition, the CMV promoter of pcDNA5/FRT was exchanged with the human *eukaryotic translation elongation factor 1 alpha 1* (*EEF1A1*) promoter to prevent epigenetic silencing^15,16^. Primers were designed using Geneious v8.1 (Biomatters Ltd) with a 20 bp homologous overhang and matching melting temperatures. Amplification of the target sequences was achieved with AccuPrime Pfx SuperMix (Thermo Fisher Scientific) according to manufacturer’s instructions. Subsequently, the PCR fragments were cleaned up using agarose gel electrophoresis and extracted using the QIAquick Gel Extraction kit (QIAgen). All cloning steps were performed via In-Fusion cloning (Takara Bio Inc.) following manufacturer’s instructions. Briefly, 50 ng of linearized vector was incubated with the inserts at molar ratio 1:2 in presence of the In-Fusion enzyme at 50 °C for 15 min. Then, 2.5 µl of the Infusion reaction was transformed into Stellar Competent Cells (Takara Bio Inc.) heat-shocked and plated onto agar plates containing appropriate antibiotics. Individual clones were picked, inoculated overnight in LB broth followed by plasmid extraction using QIAprep Spin Miniprep kit (Qiagen) according to manufacturer’s instructions. Subsequently the plasmids were analyzed for correct inserts using restriction digestion and Sanger sequencing. Stable CD33M-DAP12, CD33^∆E2^-DAP12, CD33M-DAP12-GCaMP6m and CD33^∆E2^-DAP12-GCaMP6m reporter cell lines were generated by lipofectamine transfection (Invitrogen) of plasmids into Flp-In-293 cells according to manufacturer’s instructions with a 1:9 molar ratio of pcDNA5/FRT to pOG44 plasmid. 48 h post transfection the cells were split to 25–30% confluency. The following day, the culture medium was exchanged to 293 medium containing 150 µg/ml Hygromycin B to select for stable transfected clones. Approximately 20 clones per construct were then picked and expanded. When the clones reached an appropriate number of cells, they were examined for transgene expression by flow cytometry. For positive tested clones, two subclonal dilution steps were performed to ensure monoclonality and isogeneticity. Retested clones for the generated cell lines were then used to perform experiments.

### Generation of TREM2 reporter cell line

The HEK-293/NFAT-NLucP cell line was stably transfected with the constructs DAP12 (Gene ID 7305), or TREM2 (Gene ID 54209) plus DAP12. In order to have single clones, one round of limiting dilution was performed, in which the cells were seeded at a very low cell density. After qPCR analysis, HEK-293/NFAT-NLucP/TREM2 + DAP12 K9 clone (hereinafter referred as TREM2 + DAP12) was identified as the final target clone, and HEK-293/NFAT-NLucP/DAP12 K1 clone (hereinafter referred as DAP12) as control.

### Detection of extracellular CD33 expression by flow cytometry

The cells were seeded 48 h prior experiment in 6-well plates at a density of 5 × 10^5^ cells per well. For the staining procedure, the cells were washed three times with PBS and detached using a cell lifter. Subsequently, the samples were incubated with the CD33 antibody clones 1c7/1 (Cedarlane), WM53 (Abcam) or P67.6 (Santa Cruz) in PBS (all 5 µg/ml) for 1 h on ice followed by 30 min incubation with the secondary antibody PerCP/Cy5.5-conjugated (BioLegend) or PE-conjugated (Invitrogen) anti-mouse at 5 µg/ml in PBS in darkness on ice. IPSdMiG were pre-incubated with FcR Blocking Reagent human (Miltenyi) in PBS for 15 min on ice followed by incubation with anti-CD33 (5 µg/ml, clone HIM3-4, Exbio) for 1 h in darkness on ice followed by incubation with secondary antibody Alexa Fluor 647-conjugated goat-anti-mouse IgG (2 µg/ml, Jackson ImmunoResearch). Fluorescence intensity was measured by flow cytometry (BD Calibur/Accuri C6 Plus) and analyzed using FlowJo v10.

### SYK activation in CD33 and TREM2 reporter cell lines

Activation of the kinase SYK was measured as SYK phosphorylation using the AlphaLISA SureFire Ultra p-SYK (Tyr525/526) Assay Kit (PerkinElmer). Constitutive CD33-DAP12 expressing cell lines were seeded (1–2 × 10^4^ cells/well) in poly-D-lysine 384-w plate (TwinHelix, #4332) in 293 medium. At 24 or 48 h after seeding, the cells were treated as indicated and thus processed following the manufacturer’s instructions. The AlphaLISA signal was analyzed on PheraStar FSX reader (BMG).

### Calcium imaging in CD33 reporter cell lines

For calcium imaging constitutive CD33-DAP12-GCaMP6m expressing reporter cell lines were seeded in poly-L-lysine (PLL) coated 96-well µ-plates (ibidi) at a density of 2 × 10^4^ cells per well in 293 medium. At 48 h after seeding the cells were prepared for imaging by staining with Hoechst 33342 (5 µg/ml, Thermo Fisher Scientific) in HBSS (Gibco) for 10 min at 37 °C. Afterwards, the staining solution was exchanged to the imaging solution HBSS + 1% FBS for calcium imaging.

Images were taken using the IN Cell Analyzer 2200 system (GE Healthcare Life Sciences) with a Nikon 20X, numerical aperture 0.45, Plan Fluor, ELWD, Corr Collar 0–2.0, CFI/60 objective with one by one binning and the polychroic changer set to QUAD1. The signal of the GCaMP6m from the CD33-DAP12-GCaMP6m expressing cells was collected as images for 95 s with ƒ = 1 Hz. Brightfield and Hoechst 33342 images were only taken at the beginning and the end. Antibodies (see Table 1) were automatically added to the cells after 5 s of baseline imaging. Images were analyzed using FIJI for ImageJ and calcium signal was calculated using the *ΔF/F(t)* equation^33^. For statistical analysis the area under curve (AUC) and the maximum *ΔF/F(t)* signal were calculated for each antibody in each individual experiment.

### Generation of iPSC-derived microglia

BIONi induced pluripotent stem cell (iPSC) lines (isogenic control BIONi010-C, CD33^−/−^ knockout BIONi010-C-9, CD33^∆E2^ variant BIONi010-C-5) were generated and kindly deposited by Janssen Pharmaceutica (commercially available at EBiSC, European Bank for induced pluripotent Stem Cells, https://cells.ebisc.org) and were cultured on geltrex-coated six-well plates in TeSR-E8 (STEMCELL) medium with a complete medium change every 24 h. For the differentiation into iPSC-derived microglia-like cells (iPSdMiG) BIONi010-C, BIONi010-C-9 and BIONi010-C-5 iPSCs were detached when reaching 70–80% confluency using 1 mg/ml collagenase IV (Thermo Fisher Scientific) for 30 min at 37 °C. The colonies were collected carefully in DMEM/F-12 (Gibco) and pelleted by gravity. The supernatant was aspirated and the colonies were transferred onto non-coated petri culture dishes to allow formation of embryoid bodies (EBs). The differentiation protocol was carried out according to the proprietary protocol at the LIFE & BRAIN GmbH (EP20162230). The iPSdMiG were produced from 4 to 6 weeks of differentiation and harvested from the supernatant during the following 7-week peak production phase. Harvested iPSdMiG were plated onto poly-L-lysine-coated culture dishes and experiments were performed 24 h after plating.

### Immunocytochemical analysis of iPSdMiG

For the immunocytochemical analysis 2 × 10^4^ iPSdMiG were seeded per well of a PLL-coated 96-well µ-plate (ibidi). The next day, iPSdMiG were fixed with 4% paraformaldehyde (PFA, Alfa Aeser) for 10 min at room temperature. Afterwards, iPSdMiG were blocked for 1 h in blocking solution containing 10% FBS for surface markers, and 10% FBS with 0.1% Triton X-100 (Sigma Aldrich) for cytosolic proteins at room temperature. Primary antibodies rat-anti-human CD11b (2.5 µg/ml, BD Biosciences, #553308), APC-conjugated mouse-anti-human CD45 (1:100, BD Biosciences, #555485), mouse-anti-human CD64 (2 µg/ml, Santa Cruz, #SC-1184), mouse-anti-human CD68 (1:200, Dako, #M0718), Alexa Fluor 488-conjugated mouse-anti-human CX3CR1 (1 µg/ml, Santa Cruz, #SC-377227), rabbit-anti-human IBA1 (1 µg/ml, Synaptic Systems, #234–003), rabbit-anti-human P2RY12 (1 µg/ml, Sigma Aldrich, #HPA014518), rabbit-anti-human PU.1 (1:100, Cell signaling, #2258) and rabbit-anti-human TMEM119 (1 µg/ml, Abcam, #185333) were incubated in respective blocking solutions over night at 4 °C. After incubation, the cells were washed and incubated with respective secondary antibodies Alexa Fluor 488-conjugated goat-anti-mouse or rabbit IgG (4 µg/ml, Thermo Fischer, #A11001, #A11008), Alexa Fluor 555-conjugated goat-anti-rabbit IgG (4 µg/ml, Thermo Fischer, #A21429) or Cy3-conjugated goat-anti-rat IgG (2 µg/ml, Jackson ImmunoResearch, #112-166-072) in blocking solution for 90 min at room temperature. Nuclei staining was performed using 4’-6’ diamidino-2-phenylindole (DAPI, Sigma Aldrich). Images were taking using a Leica DMI 6000B fluorescence microscope.

### Semi-quantitative real-time polymerase chain reaction (qPCR) analysis

RNA was isolated from iPSdMiG using the standard chloroform-phenol method. The cells were incubated with QIAzol (Qiagen) and chloroform (Roth) and centrifuged. The RNA-containing upper phase was extracted and incubated with an equal volume of isopropanol (Roth) for at least 2 h at − 20 °C. Finally, the RNA was centrifuged and the pellet was washed three times with 70% ethanol (Roth) and resolved in RNase-free DEPC-treated water. RNA was transcribed with the superscript III reverse transcriptase kit (Invitrogen) following manufacturer’s instructions. *CD33M* and *CD33*^*∆E2*^ gene transcript levels were then analyzed by qPCR using isoform-specific primers (CD33M forward: 5′-GCTGTGGGCAGGGGC-3′, CD33M reverse: 5′-CCTTCCCGGAACCAGTAACC-3′, CD33^∆E2^ forward: 5′-CCCTGCTGTGGGCAGACTTG-3′, CD33^∆E2^ reverse: 5′-GCACCGAGGAGTGAGTAGTCC-3′). *Glyceraldehyde 3-phosphate dehydrogenase* (*GAPDH*, forward: 5′-CTGCACCACCAACTGCTTAG-3′, reverse: 5′-TTCAGCTCAGGGATGACCTT-3′) was used as house-keeping gene. 200 ng of cDNA was used as input together with SYBR Green PCR Master Mix (Applied Biosystems) and respective gene-specific primers. Amplification and detection were performed on an ABI 5700 Sequence Detection System (PerkinElmer) as follows: 95 °C, 10 s; 40 cycles of 95 °C for 15 s, 60 °C for 30 s and 72 °C for 30 s. Relative gene transcription was quantified using the *ΔΔCT* method with GAPDH as internal control.

### SYK activation in iPSdMiG

SYK activation in iPSdMiG was determined as the ratio of phosphorylated SYK over total SYK. Therefore, 2 × 10^4^ iPSdMiG were seeded per well of a PLL-coated 96-well plate (Corning). After 24 h the cells were co-stimulated for 5 min with anti-TREM2 (5 µg/ml, R&D Systems, see Table 2) and respective CD33 antibodies or isotype controls (10 µg/ml, see Table 1). Thereby, a new P67.6 full-length antibody (BioLegend) was used as high endotoxin contamination was measured for the P67.6 Santa-Cruz antibody and possible effects of endotoxins on phosphorylation of SYK in iPSdMiG could not be excluded. pSYK levels were detected by the AlphaLISA SureFire Ultra p-SYK (Tyr525/526) Assay Kit (PerkinElmer) and normalized to the values of total SYK using the AlphaLISA SureFire Ultra Total SYK Assay Kit (PerkinElmer) according to the 2-plate assay protocol for adherent cells. All samples were measured as technical duplicates in 384-well OptiPlates (PerkinElmer). The plate was measured using the standard AlphaLISA settings on a PerkinElmer EnVision 2104 system. For the analysis, the technical duplicates were averaged and the pSYK signal was normalized to the total SYK signal. Data was displayed as pSYK/tSYK relative to the control anti-TREM2/IgG1 (anti-CD33 Ctrl).

### PHrodo *S. aureus* BioParticle phagocytosis in iPSdMiG

IPSdMiG were seeded at a density of 2 × 10^4^ cells per well of a 96-well µ-plate (ibidi) as described above. Subsequently, iPSdMiG were incubated with 0.25 mg/ml pHrodo Green *Staphylococcus aureus* BioParticles (Invitrogen) for 60 min at 37 °C together with 10 µg/ml of the respective CD33 antibodies or the isotype controls (see Table 1). Afterwards, the cells were washed, counterstained with Hoechst 33342 (5 µg/ml, Invitrogen) for 10 min at 37 °C and analyzed by live cell imaging using an IN Cell Analyzer 2200 system (GE Healthcare). The image intensity was measured by Image J version 1.53 h. The background intensity was subtracted from the image intensity and then normalized to the isotype control.

### Ethics approval and consents to participate and publish

Ethics approval and consent to participate for generating the iPSC lines and their use for academic research were obtained by EBiSC.

### Statistical analysis

Calcium imaging and iPSdMiG results were presented as mean + SEM and analyzed using SPSS v22 (IBM) as indicated. Briefly, the data was checked for normal distribution by Shapiro–Wilk test and for equality of variances by Levene’s test prior to analysis. Subsequently, unless otherwise stated Welch-ANOVA with Games-Howell post hoc was used as equality of variances could not be guaranteed. Statistical analysis of pSYK CD33M-DAP12 reporter cell line data was performed using the GraphPad Prism 6.0.0 software (GraphPad Software, San Diego, CA, USA). Data were calculated as mean ± or + S.D. and analyzed for statistical significance by using the one-way ANOVA followed by Dunnett’s test. P values less than 0.05 were considered statistically significant.

### Schematic drawings and generation of figures

Servier Medical ART: SMART was used to create the schematic drawing in Figs. 1a + b and 2a under the license agreement creativecommons.org/licenses/by/3.0/deed.en. Graphs were created using GraphPad Prism 6.0.0 (GraphPad Software, San Diego, CA, USA) and assembled using CorelDRAW Graphics Suite 2019 (Corel Corporation, Ottawa, Canada).

## Supplementary Information

Supplementary Information.
