# Supplementary material for: Reporter cell assay for human CD33 validated by specific antibodies and human iPSC-derived microglia
Source: Sci Rep. 2021 Jun 29;11:13462. doi: 10.1038/s41598-021-92434-2 (PMC8242067; doi:10.1038/s41598-021-92434-2)
Supplement: Supplementary file 1 — Supplementary Information. [file 41598_2021_92434_MOESM1_ESM.pdf]

Supplementary material

Reporter cell assay for human CD33 validated by specific antibodies and human iPSC-derived microglia

Jannis Wißfeld[1], Mona Mathews[1,4], Omar Mossad[1], Paola Picardi[2], Alessandro Cinti[2], Loredana Redaelli[2], Laurent Pradier[3], Oliver Brüstle[1,4], Harald Neumann[1*]

[1]Institute of Reconstructive Neurobiology, University of Bonn Medical Faculty and University Hospital Bonn, Venusberg-Campus 1, 53127 Bonn, Germany

[2]Axxam SpA, Via Meucci 3, 20091 Bresso, Italy

[3]Sanofi, 1 Av P. Brossolette, 91380 Chilly-Mazarin, France

[4]LIFE & BRAIN GmbH, Cellomics Unit, Venusberg-Campus 1, 53127 Bonn, Germany

*** Corresponding author:**

Harald Neumann, harald.neumann@uni-bonn.de, Tel: +49-228-6885-500, Fax: +49-228-6885-501

Supplementary figures

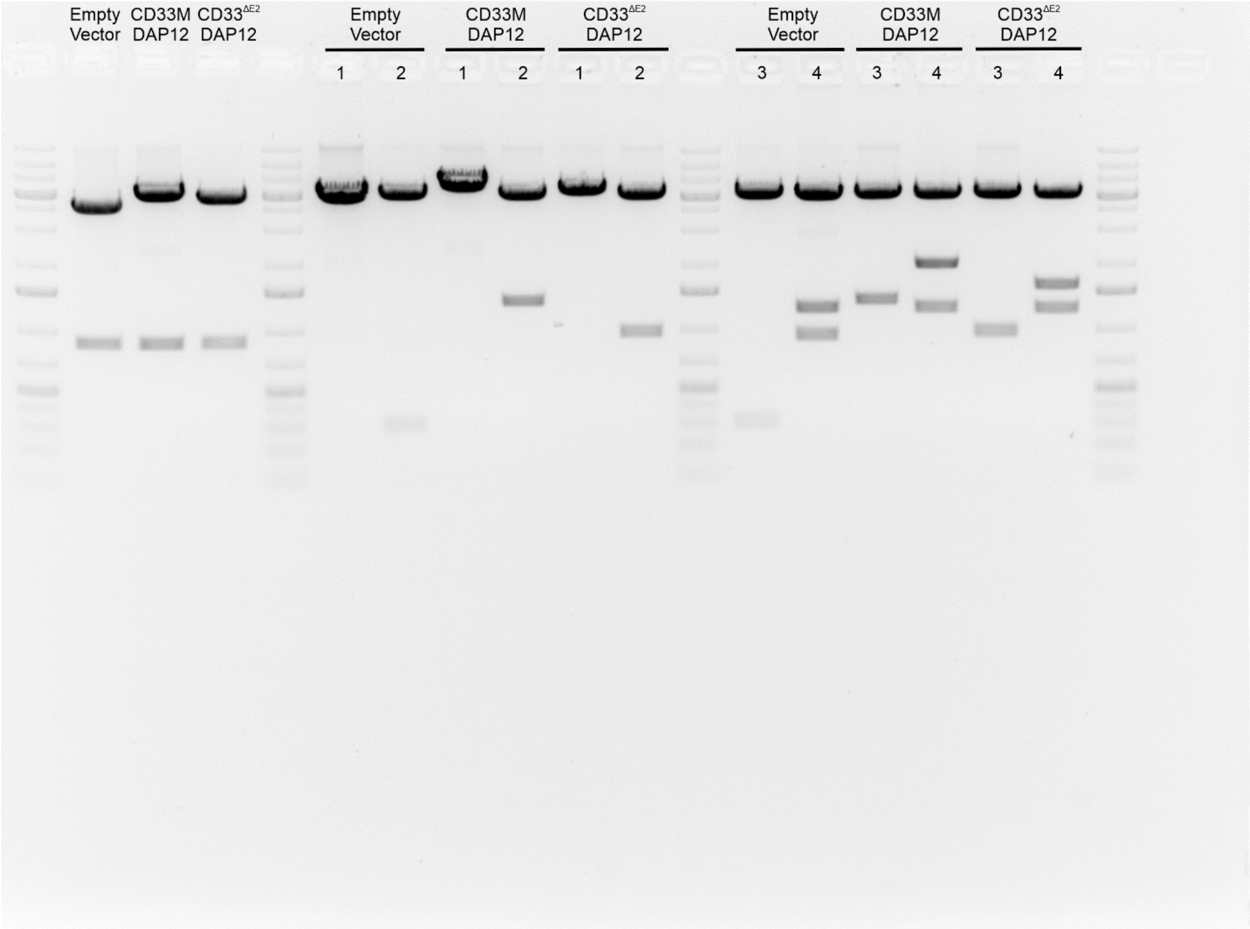

Supplementary Figure 1: Full gel image of CD33 reporter cell line constructs. Gel electrophoresis image of CD33-DAP12 constructs cloned into pcDNA5/FRT after digestion by EcoRI (lanes 2-4 (DNA ladder in lane 1). Successful exchange of the viral CMV promoter with the human *EEF1A1* promoter was indicated by a second band after digestion with XhoI at 1339 bp (CD33M) or 958 bp (CD33$^{\Delta E2}$; lanes 6-11). 1 marks the CMV-bearing plasmid and 2 marks the *EEF1A1*-containing plasmid. Gel electrophoresis image of pcDNA5/FRT-CD33-DAP12-GCaMP6m plasmids after digestion with XhoI. GCaMP6m positive clones exhibited three bands compared to the control with only two bands (lanes 13-18). 3 marks the construct without IRES-GCaMP6m motif and 4 marks the constructs with IRES-GCaMP6m motif. As DNA ladder the GeneRuler 1 kb Plus DNA Ladder (Thermo Scientific) was used in lanes 1, 5, 12 and 19.

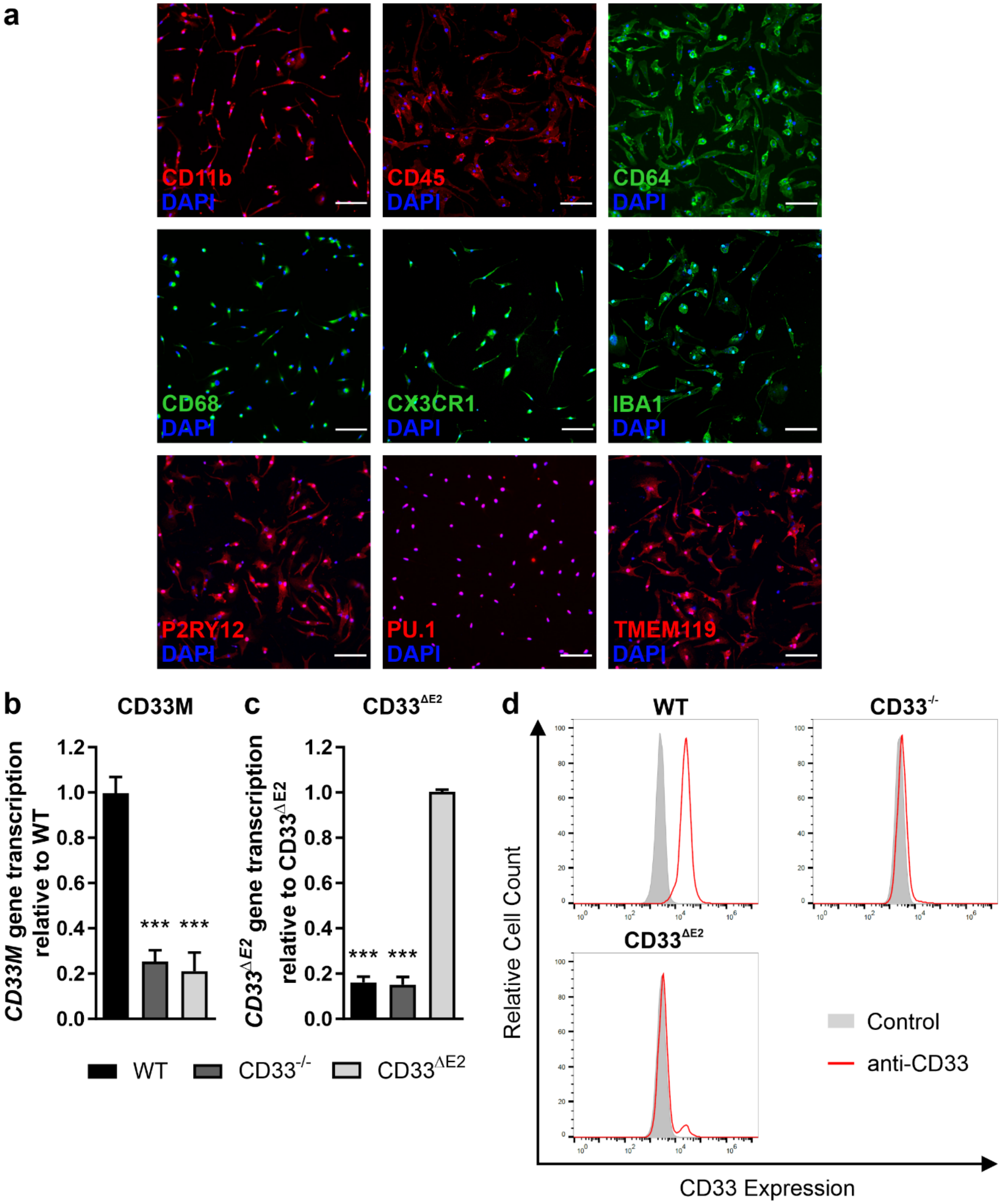

Supplementary figure 2: Characterization of iPSdMiG. (a) IPSdMiG expressed lineage-specific markers CD11b, CD45, CD64, CD68, CX3CR1, IBA1, P2RY12, PU.1 and TMEM119. Scale bar: 100µm. **(b)** Gene transcription of full-length CD33M assessed by qPCR. $CD33^{-/-}$ and $CD33^{\Delta E2}$ iPSdMiG exhibited a sharply decreased CD33M gene transcription compared to WT iPSdMiG. Data are presented mean + SEM; n = 3-4; *** $p \leq 0.001$ compared to WT iPSdMiG determined by ANOVA followed by Bonferroni post hoc test. **(c)** Gene transcription of the isoform $CD33^{\Delta E2}$ assessed by qPCR. $CD33^{\Delta E2}$ iPSdMiG exhibited a sharply elevated $CD33^{\Delta E2}$ gene transcription compared to WT and $CD33^{-/-}$ iPSdMiG. Data are presented mean + SEM; n = 3; *** $p \leq 0.001$ compared to $CD33^{\Delta E2}$ iPSdMiG determined by ANOVA followed by Bonferroni post hoc test. **(d)** CD33 surface expression analyzed by flow cytometry revealed absent or sharply decreased CD33 expression in $CD33^{-/-}$ and $CD33^{\Delta E2}$ iPSdMiG compared to WT iPSdMiG.
